# A Standard of Sanity

**Published:** 1890-05-10

**Authors:** 


### The Hospital

A Weekly Journal of

**Science, Medicine, Nursing, and Philanthropy.**

*For Hospitals, Asylums, Medical Practitioners, Students, Nurses, Families, Parishes, Congregations, and the Charitable Public.*

Vol. VIII.—No. 189.] [May 10, 1890.

### A Standard of Sanity.

Science is gradually preparing for us a standard of sanity, a standard which will be reasonably simple, and capable of application to individual cases. Lawyers, as well as doctors, know well how valuable such a standard would be if actually available; how much distress it would prevent, and how greatly it would facilitate business of various kinds. A man was brought before one of the Metropolitan magistrates the other day and charged with cruelly treating his only child, a girl of some twelve or fourteen years of age. The evidence showed that the man was very unlike ordinary fathers in his way of dealing with the little girl. He used to attack her at odd times, without any reason, with a solid square of wood, and to strike such heavy blows about her head and face as to cause serious injuries. Sometimes, equally without reason, he would leave her alone for considerable periods. Such conduct, now brutal, and now indifferent, was a puzzle both to the neighbours and to the magistrate. But a light was thrown upon it by a gentleman in the court, who stepped forward at the close of the hearing and whispered to the magistrate that the accused was "as mad as a March hare." He, himself, had been in business with him, and had been brought to ruin by his vagaries. The magistrate, the police, and the neighbours all concurred at once in the madness theory. Why was there such an immediate consensus of opinion on what is often considered an extremely difficult question to decide? The man had shown two very marked peculiarities; an unnatural want of kindness towards his own offspring; and a want of purpose in his conduct quite unusual in the average human being. In this case the combination of two peculiarities, viz., the lack of ordinary human feelings of kindness and the concomitant defect in purposed intelligence, constituted for the magistrate, the police, and the audience an obvious standard of sanity which they applied, with conviction, on the spot. No man could behave as the prisoner had behaved without thereby proving himself insane.

Now let the reader transport himself in imagination from the Metropolitan police court to the cold and arid wastes of Siberia. There is a large building standing in grim solitude in the midst of treeless fields. One of the occupants of the building is a young lady of twenty-seven, little more than a girl, though married. She is of middle height, neat as to her personal appearance, with brown hair and eyes, and a pleasing, honest, and vivacious expression. She is accomplished as well as clever and original. Not only has she taken certificates and honours at her school examinations, but she is of a fresh and independent mind, and can think for herself. She belongs to that class who in this country are alternately patronised, smiled at, and admired; she is, in short, one of the latest products of nineteenth century civilization—a lady politician. In England her politics and her cleverness would have made her a speaker on the milder social questions at drawing-room meetings; in Russia they have proved passports to a prison. The prison treatment of herself and other ladies who are incarcerated with her is bad, and Madam Sihida, for that is her name, takes the lead in protesting and striving to get things mended. But things grow worse instead of better, and Madam Sihida adopts the recognised Russian method of compelling attention to grievances and boxes the ears of the Governor of the prison. In England such a ridiculous offence would not be possible, because reasonable complaints receive reasonable attention. But even if such an absurd insult were offered by a young lady to a grown-up man and a gentleman, it would be considered quite sufficiently punished by a twenty-four hours' sojourn in a silent cell. What happens in Russia? This young lady is seized by brawny men, dragged to a long narrow bench, stripped of every article of clothing until she is quite naked in the men's presence, thrown down on her face on the bench, two or more of the men hold her legs or sit on them, two or more hold fast her arms, another takes a "plet," and, beginning at the neck, traverses the whole length of the naked back to the buttocks with well-directed blows. A hundred such blows fall one by one with slow monotony, and then this tender and delicate young lady is at liberty to rise and seek her cell. She does not rise; she does not move; she is dead.

What would you like to do, English woman or English man who read this, to the half dozen human creatures who carried out so terrible and incredible a murder? What would you like to do to the Governor of the prison who ordered the sentence? What would you like to do to the government, consisting of educated men, of nobles, of princes, of archbishops, and of a most Christian Emperor, who make the laws, whose administration ends in such a tragedy? Would you like to be content with flinging hard words at them? Hard words lead to no result. If you, English woman, were in the place of God, and had the power of God, you would pronounce the solemn doom of death and of eternal judgment upon every one of them. Such things are not uncommon under Russian rule. Young women who have not yet come to sober age, but are still half children; young men, mere boys, and University students, who play at politics, are seized in their homes, sometimes on mere suspicion, and, without trial or an opportunity of defence, are hurried off "by administrative order" to prisons thousands of miles away; prisons foul and pestilential; prisons ruled and managed by creatures who look like men and have the cunning of men, but whose hearts are so cruel that not even devils could be worse, and whose intelligence is so blunted that no savage beast in its hunger could be less amenable to argument or persuasion. We are not recounting Russian atrocities; we do not need to do so; the newspapers have been full of them for weeks past. We merely give a sample by way of illustrating our view of the present condition of Russia and its people and affairs.

It is impossible to account for the making of such laws as disgrace the Russian statute book; for the support of such laws by archbishops and bishops, princes, nobles, and the government generally; for the administration of such laws by officers and men of the army and the police force; for the quiet submission to such laws by the middle classes and the population generally—we say it is impossible to account for all these things except on this one hypothesis—that the Russian nation is insane. From the Emperor and the government, down through all ranks, and to the lowest classes of the people, the noblest characteristics of humanity have been destroyed; and what is left in each case is an imbecile and brutalised caricature, neither man nor beast. If this be considered a strange statement let the facts be taken into account. Russia is a Christian nation, the most Christian of all the Christian nations. She prides herself above all things on the orthodoxy and purity of her Christian faith. But Christianity is either something or nothing. Is it something or is it nothing? And if it be something—what is it? It is a way of living; and it is a way of living which all its loyal followers agree to carry out in daily practice. It may be more than this; but at least, and undeniably, it is this. The way of living for all Christian men is to love all other persons as well as they love themselves. That is the irreducible minimum. We are almost ashamed to ask our readers to compare the way of thinking and acting practised by the Russian Emperor, by the Government, by the archbishops, bishops and clergy, by the army and by the police force, with that simple element of their faith which tells them to do to all other people exactly as they would be done by. How can such a practice coexist with such a creed except on the one hypothesis that the persons who combine in themselves both the creed and the practice are mad? They have not the brains to perceive that the creed and the practice are mutually destructive. Their conduct is in so many departments so far removed from that of what may be called normal humanity that no other hypothesis can possibly account for it but that of insanity. If it be argued that no man or nation can become insane except as the result of the operation of adequate causes, the answer is that ages of irresponsible despotism on the part of the Russian Emperors; that the sharing in such despotism for ages of princes, nobles, ecclesiastics, soldiers and police; and that the submission to such despotism for ages on the part of the people, have gradually but surely impaired the mental energy of all classes, until at last a true and proper national insanity is the result. The causes we maintain have been quite adequate for the production of the effect.

Is it asked what may be the duty of other nations supposing our view to be simply and scientifically correct? What is the duty of a more limited population, say of a town or village, which has a dangerous and murderous madman in its streets? The duty is to secure the lunatic in order that he may do no more harm either to others or himself. The time it may be hoped will come, and that before long, when governments that are wickedly and criminally insane will be laid hands upon by a combination of other governments, and destroyed utterly and finally. The time is long since past when for the sake of a few mad rulers great nations of people tamely submitted to have their freedom destroyed and their manhood abused and ruined.

